# Reviewed article: Vieria KM, Menezes VC, Santos ACC, Franco ALMM, Carvalho FM, Faccini LS, Iser BPM. Orofacial clefts in newborns in Brazil: a time series study, 2010-2021. Epidemiol Serv Saude. 2025:34;e20240027.

**DOI:** 10.1590/S2237-96222025v34e20240027.a

**Published:** 2025-05-12

**Authors:** Kellyn Kessiene de Sousa Cavalcante

**Affiliations:** Universidade Federal do Ceará, Saúde Comunitária

**Table:** 

Rating	Excellent	Good	Average	Below average	Poor
Interest			✓		
Quality		✓			
Originality				✓	
Overall		✓			

**Table:** 

Questionnaire	Yes	No	Not applicable
Does the manuscript contain new and significant information to justify publication?	✓		
Does the Abstract (Summary) clearly and accurately describe the content of the article?	✓		
Is the problem significant and concisely stated?	✓		
Are the methods described comprehensively?	✓		
Are the interpretations and conclusions justified by the results?	✓		
Is adequate reference made to other work in the field?	✓		

## Manuscript Structure:

Length of article is: Adequate

Number of tables is: Adequate

Number of figures is: Adequate

Please state any conflict(s) of interest that you have in relation to the review of this paper (state “none” if this is not applicable): None

## Comments

Sugiro melhorar os métodos, transferir algumas informações para a introdução; e organizar melhor a estrutura das informações desse tópico.

